# The Emerging Applications of Nanotechnology in Neuroimaging: A Comprehensive Review

**DOI:** 10.3389/fbioe.2022.855195

**Published:** 2022-07-06

**Authors:** Khunza Faiz, Fred C. Lam, Jay Chen, Ekkehard M. Kasper, Fateme Salehi

**Affiliations:** ^1^ Department of Radiology, McMaster University Faculty of Health Sciences, Hamilton, ON, Canada; ^2^ Koch Institute for Integrative Cancer Research at MIT, Cambridge, MA, United States; ^3^ Division of Neurosurgery, Saint Elizabeth Medical Center, Brighton, MA, United States

**Keywords:** nanotechnology, neurooncology, neuroimaging, neurodegenerative diseases, cerebrovascular disease

## Abstract

Neuroimaging modalities such as computer tomography and magnetic resonance imaging have greatly improved in their ability to achieve higher spatial resolution of neurovascular and soft tissue neuroanatomy, allowing for increased accuracy in the diagnosis of neurological conditions. However, the use of conventional contrast agents that have short tissue retention time and associated renal toxicities, or expensive radioisotope tracers that are not widely available, continue to limit the sensitivity of these imaging modalities. Nanoparticles can potentially address these shortcomings by enhancing tissue retention and improving signal intensity in the brain and neural axis. In this review, we discuss the use of different types of nanotechnology to improve the detection, diagnosis, and treatment of a wide range of neurological diseases.

## Introduction

Current non-invasive modalities used in neuroimaging and diagnosis of diseases of the central nervous system (CNS) include: computed tomography (CT), magnetic resonance imaging (MRI), functional MRI (fMRI), functional near-infrared spectroscopy, functional photoacoustic imaging, and molecular imaging including positron emission tomography (PET), single photon emission CT (SPECT), and molecular MRI (George et al., 2018). While conventional CT and MRI are by far the workhorses in neuroradiology, limitations of these techniques exist, including: the relative low sensitivity and tissue resolution of CT, the long acquisition time required for MRI, and the high cost of radioisotopes and low resolution of PET (George et al., 2018). Furthermore, common gadolinium-based (Gd) contrast agents such as gadopentetate dimeglumine (Magnevist^®^, Bayer), administered intravenously to allow for enhancement and better visualization of both intra- and extra-axial brain tumors, have a short half-life in the bloodstream, low retention time in the tumor microenvironment, and increased risk of nephrotic systemic fibrosis in patients with renal insufficiency (Bayer, 2017). To overcome these limitations, researchers have began incorporating the use of nanotechnology to enhance the ability of these agents to achieve longer retention time in the CNS, better spatial resolution on imaging, and delivery of therapies to the cells of interest in the brain (Ngowi et al., 2021).

The diverse biophysical and chemical properties of nanoparticles (NPs) are distinct advantages for their use in CNS applications. Their small size (usually 1–100 nm in largest diameter), modularity, diverse chemical compositions, and ability to be functionalized with surface moieties to allow for targeting across the blood-brain barrier (BBB) to a specific cell type of interest, makes NPs an attractive carrier for the delivery of cargo into the CNS for use in downstream biomedical applications (Sim and Wong, 2021). In this review, we will discuss the emerging use of nanotechnology to assist in the imaging of neurological conditions including: 1) Primary and secondary brain tumors; 2) Cerebrovascular disorders; 3) Neurodegenerative disorders; and 4) Functional neurological disorders.

## Nanotechnology for the Imaging of Brain Tumors

MRI with and without the use of Gd contrast allows for the delineation of higher grade primary brains tumors such as glioblastoma, secondary brain metastases, and extra-axial tumors such as meningiomas, due to the nature of these tumors to secrete vascular endothelial growth factor which causes the formation a tortuous and leaky BBB around the tumor which allows for the extravasation of contrast agent, leading to visualization of the tumor mass on subsequent T1-weighted MRI sequences (Jain et al., 2007; George et al., 2018). However, conventional contrast-enhanced MRI sequences are limited in being able to differentiate the phenomenon of pseudoprogression (caused by radiation changes to surrounding tumor vasculature increasing leakiness and subsequent enhancement on post-treatment MRI scans) from true tumor progression.

To increase the spatial resolution of tumors on MRI, magnetic NPs such as iron oxide nanoparticles (IONPs) or manganese oxide nanoparticles (MnO NPs) have demonstrated increased relaxivity on T1-and T2-weighted MRI sequences (Vallabani et al., 2019). Functionalization of IONPs and MnO NPs with folate or albumin have enabled specific targeting of glioma cells in orthotopic mouse models of glioma, enabling longer periods of signal enhancement on MRI and could possibly aid in differentiating between true tumor signal versus pseudoprogression (Chen et al., 2014; Zhou et al., 2019). Other researchers have synthesized Gd-chelated-IONPs functionalized with interleukin-13 (Li et al., 2015), antibodies against the mutant epidermal growth factor receptor (EGFRvIII) (Hadjipanayis et al., 2010), or cyclic arginine-glycine-aspartic acid (cRGD) peptides (Booth et al., 2017), to enhance glioma targeting for enhanced imaging in preclinical animal models of glioma. A recent clinical trial demonstrated the ability of the FDA-approved IONP ferumoxytol conjugated to gadolinium contrast to define glioma pseudoprogression on MRI (Barajas et al., 2019), while another pilot study demonstrated the ability of feromoxytol to detect tumor-associated macrophages on enhanced MRI (Iv et al., 2019), showing the potential for IONP enhanced MRIs to improve cellular resolution of brain tumors.

Current clinical trials investigating the use of IONPs for molecular imaging of brain tumors include: 1) A phase I safety trial of intravenous ferumoxytol for dynamic MR imaging of recurrent high-grade gliomas patients on chemotherapy (NCT00769093) (Institute, 2017a); 2) A phase I study of ferumoxytol to assess early treatment response using perfusion MRI in patients with glioblastoma (NCT00660543) (Institute, 2017b); 3) A phase 0 safety and effectiveness exploratory trial of ferumoxytol in assessing vascular properties of pediatric brain tumors in a single imaging session (NCT00978562) (Institute, 2018); 4) A phase 2 study comparing ferumoxytol in characterizing tumor vasculature compared to Gd-contrast MRI in 3 and 7 T MRI machines in patients with malignant brain tumors (NCT00659126) (Institute, 2021); 5) A phase 2 study of ferumoxytol in characterizing tumor-associated vasculature in patients with primary brain tumors or lung or breast brain metastases using MRI (NCT00103038) (Institute, 2017c); and 6) A phase 2 study of the safety of ferumoxytol in causing CNS inflammation (NCT00659776) (OHSU, 2022).

## Nanotechnology for the Diagnosis of Neurodegenerative Disorders

Neurodegenerative disorders encompass a wide variety of neurological conditions, from Alzheimer’s Disease (AD), Parkinson’s Disease (PD), Huntington’s Disease (HD), to Multiple Sclerosis (MS) and motor neuron diseases. As a full discussion of all these disorders goes beyond the scope of this review, we will focus on AD and PD—the two neurodegenerative disorders for which there have been the most prolific application of NPs as a neuroimaging tool to aid in diagnosis and management.

i) Alzheimer’s Disease

The two pathognomonic features of AD include extracellular beta-amyloid (Aβ) plaques and neurofibrillary tangles of hyperphosphorylated tau protein in dystrophic neurites (Alzheimer’s Association, 2014). Current imaging modalities for the diagnosis of Aβ plaques and tau tangles include radioactive PET tracers such as fluorodeoxyglucose, [^18^F]flutemetamol, and [^18^F]flortaucipir (Mason et al., 2010). However, these radioisotopes are costly and not readily available worldwide. To help overcome these limitations, researchers have developed various IONP nanoprobes that can detect Aβ plaques in the brain via a conjugated Aβ antibody on MRI in transgenic AD mice (Sillerud et al., 2013; Viola et al., 2015; Li et al., 2016). Nanotechnology-based PET has also been used in preclinical models to detect Aβ plaques *in vivo* (Sharma et al., 2017), demonstrating the potential for use in diagnostic imaging of human AD patients.

ii) Parkinson’s Disease

Progressive degeneration of dopaminergic neurons in the basal ganglia substantia nigra with abnormal deposition of α-synuclein protein is thought to cause the bradykinesia, and dystonia seen in PD patients (Obeso et al., 2017). Aggregates of α-synuclein seen in other nuclei in the brain are thought to further contribute to the non-motor symptoms seen in PD patients such as hyposmia and dysautonomia, making α-synuclein an attractive biomarker for tracking disease progression (Surmeier et al., 2017). ^123^I-ioflupane labelled dopamine SPECT can be used to monitor olfactory nerve dysfunction and serial MRI of basal ganglia are current tools for diagnosis and tracking of PD (Booij et al., 2001; Morbelli et al., 2020). Novel non-invasive preclinical imaging tools to detect fibrillary α-synuclein currently lack adequate sensitivity and specificity. A recent study using a dLight1 fluorescence sensor to detect dopaminergic signals in the brains of transgenic PD mice yielded promising preliminary results in being able to detect fluorescent signals in dopaminergic neurons (Patriarchi et al., 2018), while a dopamine-responsive nanoprobe that reacts in the near-infrared spectrum detected dopamine signals in preclinical models of drug abuse and PD (Feng et al., 2018), suggesting that these experimental imaging modalities and targeted nanoprobes could have potential applications in human diagnosis of PD.

## Nanotechnology for the Imaging of Cerebral Ischemia

Acute blockage of blood vessels in the brain leads to cerebral ischemia or stroke, which requires prompt diagnosis and treatment to prevent permanent ischemic injury to the areas of the brain supplied by the affected blood vessels to prevent motor or sensory deficits (Moskowitz et al., 2010). Ischemic stroke is the second most common cause of death worldwide after cardiovascular disease, with approximately 87% mortality if left untreated (Association, 2015). The ability to detect and locate the blockage and visualize the affected region of the brain with high sensitivity and spatial resolution can allow for accurate diagnosis, treatment, and serial monitoring of the treatment outcomes.

MRI and MR perfusion scans to assess cerebral blood flow are the current gold standard non-invasive modalities for stroke imaging (Marks, 2016). Similar to the use of NPs to augment contrast enhancement for the detection of brain tumor vasculature, researchers have used NPs to increase the sensitivity and cellular resolution of areas of the brain affected by ischemia. IONPs conjugated with RGD peptide targeting the αvβ3 integrin receptors on endothelium have been used to enhance imaging of collateral vessels that form in the surrounding area of ischemia (Wang et al., 2018). Another study comparing carbohydrate wrapped ferumoxytol to a Gd-based contrast agent found that ferumoxytol was able to detect cerebral blood volume at higher resolution at steady state than dynamic susceptibility contrast MR perfusion imaging using Gd (Varallyay et al., 2013). By leveraging the relative acidic environment in the ischemic penumbra, pH-responsive IONPs encapsulated in poly(β-amino ester)-poly(aminodoamine)-poly(ethylene glycol) block copolymer were released and accumulated in the ischemic region of the brain in a rat model of cerebral ischemia, enabling detection on MRI (Gao et al., 2011). A safety study of IONPs in canines and macaque monkeys demonstrated sensitive detection of ischemia on T1-weighted MR sequences in cerebral ischemia-induced animals, providing further preclinical evidence supporting their safety for use in human stroke patients (Lu et al., 2017).

CT angiography (CTA) using iodinated contrast agent provides 3-dimensional reconstructed images of large vessel occlusion. Encapsulation of CT contrast agents may facilitate enhanced delivery of imaging agents to the affected region of the brain. Polyethylene glycol (PEG) wrapped or PEGylated BaHoF_5_ nanoprobes with an average diameter of 7 nm exhibited superior enhancement properties compared to the clinical contrast agent iohexol^®^ in CTA and CT perfusion imaging of a rat model of left middle cerebral artery occlusion (Wang et al., 2015). Gold NPs have also been used due to their biocompatibility and ability to incorporate targeting, therapeutic, and imaging moieties for multimodal imaging and theranostics. Chitosan-coated gold NPs with the ability to target fibrin were used to quantitate the amount of thrombolysis in a cerebral thromboembolic model of stroke in mice following treatment with intravenous tissue plasminogen activator (tPA). Serial CT imaging detected rapid thrombolysis following tPA treatment (Kim et al., 2017).

Ultrasonography can be used to detect and image flow through blood vessels. Ultrasound contrast enhancers such as gas-filled microbubbles increase the “echogenicity” of the vessels, thereby increasing their chances to be detected using ultrasound (Sirsi and Borden, 2009). These microbubbles are composed of albumin, lipids, or polymers and can be filled with air, perfluorocarbons, or nitrogen, and can be functionalized with surface ligands for targeting (Paefgen et al., 2015). Perfluorocarbon emulsions conjugated with anti-fibrin antibodies have demonstrated the ability to visualize thrombus in a model of cerebral ischemia on ultrasound (Chen et al., 2013a).

IONPs have also been used to visualize areas of relative signal attenuation using microwave imaging in a rabbit model of cerebral ischemia as well as in a human volunteer (Hudson et al., 2019). Applying a PEG coating to the surface of IONPs can greatly increase the circulation time of these NPs in the bloodstream (Khandhar et al., 2017), enabling three-dimensional angiography following a single injection of PEGylated IONPs to allow for real-time imaging of cerebral vasculature in mouse models to assess cerebral hemorrhage in a mouse model of traumatic brain injury over a period of hours (Goodwill et al., 2012; Orendorff et al., 2017). Taken together, these emerging uses of NPs to enhance multimodal imaging and assessment of cerebral ischemia may hopefully translate into the clinic to improve patient outcomes. Please refer to an excellent review on the emerging uses of nanotechnology in the imaging of stroke by Kaviarasi and colleagues for further information on this topic (Kaviarasi et al., 2019) and for ease of reference for our readers, we have compiled a list of NPs that are in human clinical trials or have been approved for neurological disorders (Table 1).

**TABLE 1:** Nanoparticles approved or currently in clinical trials for neurological diseases. List of nanoparticles that are in human clinical trials or have been approved for the treatment of neuro-oncologic, neurodegenerative, and cerebovascular disorders.

**Neuro-oncology**		
**Trade name**	**Product formulation**	**Level of clinical development and treatment applications**
SGT-53 (Anselmo and Mitragotri, 2016)	Transferrin-functionalized liposomal plasmid wild-type p53 DNA	NCT02340156—Phase II open label, single-arm, multicenter study of IV SGT-53 and oral temozolomide in patients with confirmed recurrent glioblastoma or progression.
DepoCyt (Zhang et al., 2008; Khanbabaie and Jahanshahi, 2012)	Liposomal cytarabine	Approved for treatment of malignant lymphomatous meningitis.
DaunoXome (Zhang et al., 2008; Khanbabaie and Jahanshahi, 2012)	Liposomal daunorubicin	Approved for HIV-related Kaposi’s sarcoma.
Neulasta (Gabizon et al., 2006; Kumar et al., 2013)	PEGylated granulocyte colony-stimulating factor	Approved for treatment of chemotherapy-associated neutropenia.
Cornell Dots (Anselmo and Mitragotri, 2016)	PEGylated silica NPs of^124^I-cRGDY NIR fluorophore	Phase 0 safety trial of imaging of melanoma and malignant brain tumors.
**Neurodegenerative Disorders**		
**Trade Name**	**Product Formulation**	**Level of Clinical Development and Treatment Applications**
Visudyne (Zhang et al., 2008; Khanbabaie and Jahanshahi, 2012)	Liposomal verteporfin	Approved for treatment of age-related mascular degeneration, pathologic myopia, and ocular histoplasmosis.
Macugen (Gabizon et al., 2006; Kumar et al., 2013)	PEGylated anti-VEGF aptimer	Approved for age-related macular degeneration.
Partisiran (Anselmo and Mitragotri, 2016)	Liposomal transthyretin siRNA	Phase I-III study for the treatment of hereditary transthyretin-mediated amyloidosis. Characterized by peripheral sensorimotor and autonomic neuropathy. RNA interference that reduces production of both wild-type and mutant transthyretin protein. Phase III APOLLO study showed significant improvement in polyneuropathy.
Copaxone (Gabizon et al., 2006; Kumar et al., 2013)	Copolymer of L-Glutamic acid, L-alanine, and L-tyrosine	Approved for the treatment of Multiple Sclerosis.
**Cerebrovascular Disorders**		
**Trade Name**	**Product Formulation**	**Level of Clinical Development and Treatment Applications**
Definity (Anselmo and Mitragotri, 2016)	Perflutren lipid microspheres	Approved ultrasound contrast agent for imaging of cerebral ischemia and transcranial brain injuries.

## Discussion

Implementation of nanoscale materials for neuroimaging is still in the early stages of preclinical development. Several limitations exist that currently prevent the widespread application of nanomaterials for use in the CNS space. The presence of the intact BBB with endothelial tight junctions that limit passage of most materials greater than 100 nm in diameter restricts the bulkiness of nanomaterials that can be delivered systemically into the brain (Sarin et al., 2009). The cellular complexity of the CNS makes it difficult to target NPs to specific cells of interest (i.e. neurons and not glia) and avoid unintended off-target effects to different types of nearby cells. Finally, the brain is bathed in cerebrospinal fluid (CSF), which pulses with each heartbeat, creating a natural washout effect that actively prevents accumulation of cargo at the desired site of delivery (Hendricks et al., 2015). Furthermore, even with targeted delivery approaches using functionalized NPs that preferentially recognize protein receptors on cell surfaces (i.e., transferrin receptors on the surfaces of glioma cells), the percent injected dose of NPs that ultimately cross the BBB via the bloodstream is meager and would require dosing human subjects with hundreds of milliliters of NPs intravenously to achieve a detectable phenotypic effect (Wilhelm et al., 2016). More invasive mechanisms of bypassing the BBB such as direct administration of NPs into the brain using stereotactic implantable catheters or use of convection enhanced delivery or local ultrasonic disruption of the BBB can improve the uptake of NPs, but these tools are limited to specialized hospital centers, require collaborative efforts from a neurosurgical team, and are generally not recommended for use in day-to-day neuroimaging procedures (McDannold et al., 2012; Hendricks et al., 2015; Magill et al., 2020).

Metallic NPs are currently the most researched nanomaterials for emerging neuroimaging technologies. Due to their physicochemical compositions, metal oxide NPs have been incorporated in a broad range of biomedical applications, from imaging contrast agents to scaffolding materials for tissue regeneration. Their small size (∼50 nm) also makes them ideal for trafficking across the BBB into the CNS space to enhance neuroimaging modalities and deliver various types of cargo for the treatment of neurological disorders (Stark et al., 1988; Jung and Jacobs, 1995; Barajas et al., 2019). The iron oxide contrast agent ferumoxytol (Feraheme^®^, AMAG) has seen increasing usage in MR imaging in North America, while the IONP formulation ferumoxtran-10 (Sinerem/Combidex) has been gaining interest in human clinical trials in Europe (Harisinghani et al., 2003; Heesakkers et al., 2008; Triantafyllou et al., 2013; Smits et al., 2016). With decades of longitudinal data regarding the biodistribution, pharmacokinetics, and pharmacodynamics of IONPs, we now have a deeper understanding of how IONPs behave in the CNS and its associated neurotoxicities, which may limit their translational potential. *In vitro* studies have demonstrated uptake of IONPs into neurons, glia, and astrocytes via micropinocytosis and clathrin-mediated endocytosis with trafficking into lysosomal compartments (Luther et al., 2013; Huerta-García et al., 2015; Pongrac et al., 2018). Exposure to silver NPs in a BBB co-culture model led to shrinkage of mitochondria, expansion of the endoplasmic reticulum, and vacuolation in astrocytes with altered gene expression across 23 genes associated with metabolic, biosynthetic, and cell death processes (Xu et al., 2013). Astrocytes increased production of reactive oxygen species and decreased viability when exposed to copper oxide NPs (Bulcke et al., 2014). Interestingly, studies have shown that the toxicity of IONPs depended on the surface coating. Polydimethyloamine coating caused cell membrane disruption and cell death in cultured cortical neurons, while aminosilane coating left the cell membrane intact but affected cellular metabolism, but dextran remained inert to cultured neurons (Rivet et al., 2012). *In vivo* toxicity experiments have demonstrated accumulation of aluminum NPs in mouse brain endothelial cells causing neurovascular damage and increased BBB permeability (Chen et al., 2013b), while acute exposure of gold NPs to male Wistar rats led to catabolic and energy metabolic suppression in the hippocampus, striatum, and cerebral cortex but long-term exposure only resulted in inhibition of catalase and energy metabolism in the cortex (Ferreira et al., 2017). Taken together, these data indicate that neurotoxicity studies need to be done in higher order organisms such as non-human primates to better understand the mechanisms of neurotoxicity associated with NPs that are applied for CNS applications in humans.

We recently reviewed emerging applications of nanotechnology in the fields of neurosurgery, neuro-oncology, and neuroradiology from the perspective of clinician-scientists (Lam et al., 2022). One emerging area is the ability to imbue NPs with theranostic capabilities—functionalizing NPs with the dual ability to detect cells of interest and deliver therapeutic cargo. A recent first-in-human trial using the EGFR antibody cetuximab conjugated to the near-infrared (NIR) fluorescent dye IRDye800 (cetuximab-IRDye800) during neurosurgical resection of gliomas demonstrated the specificity of the antibody-dye conjugate to specifically attach to EGFR-expressing glioma cells in the brain, enhancing the detection of tumor cells during surgery and potentially assisting the neurosurgeon in achieving a more extensive surgical resection, which could improve patient survival (Miller et al., 2018). Conjugation of a CD133 monoclonal antibody to a phototoxic phthalocyanine NIR dye IR700 allowed for specific targeting and imaging of CD133+ glioma stem cells and treatment using NIR photoimmunotherapy to cause tumor shrinkage and cell death in a murine intracranial orthotopic model of glioma (Jing et al., 2016). Multi-walled carbon nanotubes have been used to replace conventional silver-silver chloride electrodes used in electroencephalography to eliminate the metal-associated artifacts seen in CT and x-ray images of the brain, to allow for better visualization of the brain during image-guided neurosurgical procedures (Awara et al., 2014). Nanoknives made from silicon nitride with 20 nm tips can make minute and precise incisions to reduce the amount of cerebral tissue damage associated with using conventional neurosurgical tools (Chang et al., 2007). Finally, dextran-coated IONPs covalently linked to tPA in an agarose gel can enhance the fibrinolytic activity of tPA for days, prolonging thrombolysis and potentially improving outcomes for stroke victims (Heid et al., 2017). With further development of these exciting preclinical discoveries, there is hope that researchers will be able to bridge the gap from benchtop to bedside.

## Conclusion

The application of nanotechnology for use in neuroimaging has gained steady progress. Although this platform is still in its early stages of technology development, the ability to harness NPs as a theranostic will further enhance the function of NPs in the CNS to treat a wide variety of neurological disorders. The ability to decrease the neurotoxic nature of NPs will improve their safety profile and improve the translational potential for use in patients.

## References

[B1] Alzheimer's Association (2014). 2014 Alzheimer's Disease Facts and Figures. Alzheimers Dement. 10, e47–92. 10.1016/j.jalz.2014.02.001 24818261

[B2] Anselmo A. C. Mitragotri S. (2016). Nanoparticles in the Clinic. Bioeng. Transl. Med. 1, 10–29. 10.1002/btm2.10003 29313004 PMC5689513

[B3] Association T. A. H. (2015). Ischemic Strokes (Clots). Available at: http://www.strokeassociation.org/STROKEORG/AboutStroke/TypeofStroke/IschemicClots/Ischemic-Strokes-Clots_UCM_310939_Article.jsp#.WdzLmY-OPIU%0 .

[B5] Awara K. Kitai R. Isozaki M. Neishi H. Kikuta K. Fushisato N. (2014). Thin-film Electroencephalographic Electrodes Using Multi-Walled Carbon Nanotubes Are Effective for Neurosurgery. Biomed. Eng. Online 13, 166. 10.1186/1475-925X-13-166 25511926 PMC4290091

[B6] Barajas R. F. Hamilton B. E. Schwartz D. McConnell H. L. Pettersson D. R. Horvath A. (2019). Combined Iron Oxide Nanoparticle Ferumoxytol and Gadolinium Contrast Enhanced MRI Define Glioblastoma Pseudoprogression. Neuro Oncol. 21, 517–526. 10.1093/neuonc/noy160 30277536 PMC6422439

[B47] Bayer (2017). Magnevist Product Monograph. Available at: http://www.bayer.com/sites/default/files/2020-11/magnevist-pm-en.pdf .

[B7] Booij J. Speelman J. D. Horstink M. W. I. M. Wolters E. C. (2001). The Clinical Benefit of Imaging Striatal Dopamine Transporters with [123I]FP-CIT SPET in Differentiating Patients with Presynaptic Parkinsonism from Those with Other Forms of Parkinsonism. Eur. J. Nucl. Med. 28, 266–272. 10.1007/s002590000460 11315592

[B8] Booth H. D. E. Hirst W. D. Wade-Martins R. (2017). The Role of Astrocyte Dysfunction in Parkinson's Disease Pathogenesis. Trends Neurosci. 40, 358–370. 10.1016/j.tins.2017.04.001 28527591 PMC5462417

[B9] Bulcke F. Thiel K. Dringen R. (2014). Uptake and Toxicity of Copper Oxide Nanoparticles in Cultured Primary Brain Astrocytes. Nanotoxicology 8, 1–11. 10.3109/17435390.2013.829591 23889294

[B10] Chang W. C. Hawkes E. A. Kliot M. Sretavan D. W. (2007). *In Vivo* use of a Nanoknife for Axon Microsurgery. Neurosurgery 61, 683–692. discussion 691-682. 10.1227/01.NEU.0000298896.31355.80 17986929

[B11] Chen J. Pan H. Lanza G. M. Wickline S. A. (2013). Perfluorocarbon Nanoparticles for Physiological and Molecular Imaging and Therapy. Adv. Chronic Kidney Dis. 20, 466–478. 10.1053/j.ackd.2013.08.004 24206599 PMC4074885

[B12] Chen L. Zhang B. Toborek M. (2013). Autophagy Is Involved in Nanoalumina-Induced Cerebrovascular Toxicity. Nanomedicine Nanotechnol. Biol. Med. 9, 212–221. 10.1016/j.nano.2012.05.017 PMC3482418 22687898

[B13] Chen N. Shao C. Qu Y. Li S. Gu W. Zheng T. (2014). Folic Acid-Conjugated MnO Nanoparticles as a T1 Contrast Agent for Magnetic Resonance Imaging of Tiny Brain Gliomas. ACS Appl. Mat. Interfaces 6, 19850–19857. 10.1021/am505223t 25335117

[B14] Feng P. Chen Y. Zhang L. Qian C.-G. Xiao X. Han X. (2018). Near-Infrared Fluorescent Nanoprobes for Revealing the Role of Dopamine in Drug Addiction. ACS Appl. Mat. Interfaces 10, 4359–4368. 10.1021/acsami.7b12005 29308644

[B15] Ferreira G. K. Cardoso E. Vuolo F. S. Galant L. S. Michels M. Gonçalves C. L. (2017). Effect of Acute and Long-Term Administration of Gold Nanoparticles on Biochemical Parameters in Rat Brain. Mater. Sci. Eng. C 79, 748–755. 10.1016/j.msec.2017.05.110 28629076

[B16] Gabizon A. A. Shmeeda H. Zalipsky S. (2006). Pros and Cons of the Liposome Platform in Cancer Drug Targeting. J. Liposome Res. 16, 175–183. 10.1080/08982100600848769 16952872

[B17] Gao G. H. Lee J. W. Nguyen M. K. Im G. H. Yang J. Heo H. (2011). pH-Responsive Polymeric Micelle Based on PEG-Poly(β-Amino Ester)/(amido Amine) as Intelligent Vehicle for Magnetic Resonance Imaging in Detection of Cerebral Ischemic Area. J. Control. Release 155, 11–17. 10.1016/j.jconrel.2010.09.012 20854855

[B18] George E. Guenette J. P. Lee T. C. (2018). Introduction to Neuroimaging. Am. J. Med. 131, 346–356. 10.1016/j.amjmed.2017.11.014 29191488

[B19] Goodwill P. W. Saritas E. U. Croft L. R. Kim T. N. Krishnan K. M. Schaffer D. V. (2012). X-space MPI: Magnetic Nanoparticles for Safe Medical Imaging. Adv. Mat. 24, 3870–3877. 10.1002/adma.201200221 22988557

[B20] Hadjipanayis C. G. Machaidze R. Kaluzova M. Wang L. Schuette A. J. Chen H. (2010). EGFRvIII Antibody-Conjugated Iron Oxide Nanoparticles for Magnetic Resonance Imaging-Guided Convection-Enhanced Delivery and Targeted Therapy of Glioblastoma. Cancer Res. 70, 6303–6312. 10.1158/0008-5472.CAN-10-1022 20647323 PMC2912981

[B21] Harisinghani M. G. Barentsz J. Hahn P. F. Deserno W. M. Tabatabaei S. van de Kaa C. H. (2003). Noninvasive Detection of Clinically Occult Lymph-Node Metastases in Prostate Cancer. N. Engl. J. Med. 348, 2491–2499. 10.1056/NEJMoa022749 12815134

[B22] Heesakkers R. A. Hövels A. M. Jager G. J. van den Bosch H. C. Witjes J. A. Raat H. P. (2008). MRI with a Lymph-node-specific Contrast Agent as an Alternative to CT Scan and Lymph-Node Dissection in Patients with Prostate Cancer: a Prospective Multicohort Study. Lancet Oncol. 9, 850–856. 10.1016/S1470-2045(08)70203-1 18708295

[B23] Heid S. Unterweger H. Tietze R. Friedrich R. P. Weigel B. Cicha I. (2017). Synthesis and Characterization of Tissue Plasminogen Activator-Functionalized Superparamagnetic Iron Oxide Nanoparticles for Targeted Fibrin Clot Dissolution. Int. J. Mol. Sci. 18, E1837. [pii]10.3390/ijms18091837. 10.3390/ijms18091837 28837060 PMC5618486

[B24] Hendricks B. K. Cohen-Gadol A. A. Miller J. C. (2015). Novel Delivery Methods Bypassing the Blood-Brain and Blood-Tumor Barriers. Foc 38, E10. 10.3171/2015.1.FOCUS14767 25727219

[B25] Hudson J. S. Chung T. K. Prout B. S. Nagahama Y. Raghavan M. L. Hasan D. M. (2019). Iron Nanoparticle Contrast Enhanced Microwave Imaging for Emergent Stroke: A Pilot Study. J. Clin. Neurosci. 59, 284–290. 10.1016/j.jocn.2018.10.100 30391310

[B26] Huerta-García E. Márquez-Ramírez S. G. Ramos-Godinez M. d. P. López-Saavedra A. Herrera L. A. Parra A. (2015). Internalization of Titanium Dioxide Nanoparticles by Glial Cells Is Given at Short Times and Is Mainly Mediated by Actin Reorganization-dependent Endocytosis. Neurotoxicology 51, 27–37. 10.1016/j.neuro.2015.08.013 26340880

[B27] Institute O. K. C. (2017). Assessing Dynamic Magnetic Resonance (MR) Imaging in Patients with Recurrent High Grade Glioma Receiving Chemotherapy (Code 7228). Available at: http://clinicaltrials.gov/ct2/show/NCT00769093 .

[B28] Institute O. K. C. (2021). Ferumoxytol and Gadolinium Magnetic Resonance Imaging (MRI) at 3T and 7T in Patients with Malignant Brain Tumors. Available at: http://clinicaltrials.gov/ct2/show/NCT00659126 .

[B29] Institute O. K. C. (2018). Imaging Vascular Properties of Pediatric Brain Tumors Using Ferumoxytol and Gadolinium in a Single Imaging Session. Available at: http://www.clinicaltrials.gov/ct2/show/NCT00978562 .

[B30] Institute O. K. C. (2017). Magnetic Resonance (MR) Imaging Study Using Ferumoxytol to Assess Early Tumor Response in Patients with Glioblastoma Multiforme. Available at: http://clinicaltrials.gov/show/NCT00660543 .

[B31] Institute O. K. C. (2017). MRI Using Ferumoxytol in Patients with Primary Brain Cancer or Brain Metastases from Lung or Breast Cancer. Available at: http://clinicaltrials.gov/ct2/show/NCT00103038 .

[B32] Iv M. Samghabadi P. Holdsworth S. Gentles A. Rezaii P. Harsh G. (2019). Quantification of Macrophages in High-Grade Gliomas by Using Ferumoxytol-Enhanced MRI: A Pilot Study. Radiology 290, 198–206. 10.1148/radiol.2018181204 30398435 PMC6312434

[B33] Jain R. K. di Tomaso E. Duda D. G. Loeffler J. S. Sorensen A. G. Batchelor T. T. (2007). Angiogenesis in Brain Tumours. Nat. Rev. Neurosci. 8, 610–622. 10.1038/nrn2175 17643088

[B34] Jing H. Weidensteiner C. Reichardt W. Gaedicke S. Zhu X. Grosu A.-L. (2016). Imaging and Selective Elimination of Glioblastoma Stem Cells with Theranostic Near-Infrared-Labeled CD133-specific Antibodies. Theranostics 6, 862–874. 10.7150/thno.12890 27162556 PMC4860894

[B35] Jung C. W. Jacobs P. (1995). Physical and Chemical Properties of Superparamagnetic Iron Oxide MR Contrast Agents: Ferumoxides, Ferumoxtran, Ferumoxsil. Magn. Reson. Imaging 13, 661–674. 10.1016/0730-725x(95)00024-b 8569441

[B36] Kaviarasi S. Yuba E. Harada A. Krishnan U. M. (2019). Emerging Paradigms in Nanotechnology for Imaging and Treatment of Cerebral Ischemia. J. Control. Release 300, 22–45. 10.1016/j.jconrel.2019.02.031 30802476

[B37] Khanbabaie R. Jahanshahi M. (2012). Revolutionary Impact of Nanodrug Delivery on Neuroscience. Curr. Neuropharmacol. 10, 370–392. CN-10-370 [pii] (2012). 10.2174/157015912804143513 23730260 PMC3520046

[B38] Khandhar A. P. Keselman P. Kemp S. J. Ferguson R. M. Goodwill P. W. Conolly S. M. (2017). Evaluation of PEG-Coated Iron Oxide Nanoparticles as Blood Pool Tracers for Preclinical Magnetic Particle Imaging. Nanoscale 9, 1299–1306. 10.1039/c6nr08468k 28059427 PMC5316483

[B39] Kim D.-E. Kim J.-Y. Schellingerhout D. Ryu J. H. Lee S.-K. Jeon S. (2017). Quantitative Imaging of Cerebral Thromboemboli *In Vivo* . Stroke 48, 1376–1385. 10.1161/STROKEAHA.117.016511 28432262

[B40] Kumar A. Chen F. Mozhi A. Zhang X. Zhao Y. Xue X. (2013). Innovative Pharmaceutical Development Based on Unique Properties of Nanoscale Delivery Formulation. Nanoscale 5, 8307–8325. 10.1039/c3nr01525d 23860639 PMC3934102

[B41] Lam F. C. Salehi F. Kasper E. M. (2022). Integrating Nanotechnology in Neurosurgery, Neuroradiology, and Neuro-Oncology Practice-The Clinicians' Perspective. Front. Bioeng. Biotechnol. 10, 801822. 10.3389/fbioe.2022.801822 35223783 PMC8864069

[B42] Li S.-S. Lin C.-W. Wei K.-C. Huang C.-Y. Hsu P.-H. Liu H.-L. (2016). Non-invasive Screening for Early Alzheimer's Disease Diagnosis by a Sensitively Immunomagnetic Biosensor. Sci. Rep. 6, 25155. 10.1038/srep25155 27112198 PMC4844990

[B43] Li T. Murphy S. Kiselev B. Bakshi K. S. Zhang J. Eltahir A. (2015). A New Interleukin-13 Amino-Coated Gadolinium Metallofullerene Nanoparticle for Targeted MRI Detection of Glioblastoma Tumor Cells. J. Am. Chem. Soc. 137, 7881–7888. 10.1021/jacs.5b03991 26022213

[B44] Lu Y. Xu Y.-J. Zhang G.-b. Ling D. Wang M.-q. Zhou Y. (2017). Iron Oxide Nanoclusters for T 1 Magnetic Resonance Imaging of Non-human Primates. Nat. Biomed. Eng. 1, 637–643. 10.1038/s41551-017-0116-7 31015599

[B45] Luther E. M. Petters C. Bulcke F. Kaltz A. Thiel K. Bickmeyer U. (2013). Endocytotic Uptake of Iron Oxide Nanoparticles by Cultured Brain Microglial Cells. Acta Biomater. 9, 8454–8465. 10.1016/j.actbio.2013.05.022 23727247

[B46] Magill S. T. Choy W. Nguyen M. P. McDermott M. W. (2020). Ommaya Reservoir Insertion: A Technical Note. Cureus 12, e7731. 10.7759/cureus.7731 32432009 PMC7234073

[B4] Marks M. P. (2016). Magnetic Resonance of the Brain and Spine Vol. 2. Editor Atlas W.. (Wolters Kluwer Health).

[B48] Mason S. E. McShane R. Ritchie C. W. (2010). Diagnostic Tests for Alzheimer's Disease: Rationale, Methodology, and Challenges. Int. J. Alzheimer's Dis. 2010, 1–6. 10.4061/2010/972685 PMC2929623 20811564

[B49] McDannold N. Arvanitis C. D. Vykhodtseva N. Livingstone M. S. (2012). Temporary Disruption of the Blood-Brain Barrier by Use of Ultrasound and Microbubbles: Safety and Efficacy Evaluation in Rhesus Macaques. Cancer Res. 72, 3652–3663. 10.1158/0008-5472.CAN-12-0128 22552291 PMC3533365

[B50] Miller S. E. Tummers W. S. Teraphongphom N. van den Berg N. S. Hasan A. Ertsey R. D. (2018). First-in-human Intraoperative Near-Infrared Fluorescence Imaging of Glioblastoma Using Cetuximab-IRDye800. J. Neurooncol 139, 135–143. 10.1007/s11060-018-2854-0 29623552 PMC6031450

[B51] Morbelli S. Esposito G. Arbizu J. Barthel H. Boellaard R. Bohnen N. I. (2020). EANM Practice Guideline/SNMMI Procedure Standard for Dopaminergic Imaging in Parkinsonian Syndromes 1.0. Eur. J. Nucl. Med. Mol. Imaging 47, 1885–1912. 10.1007/s00259-020-04817-8 32388612 PMC7300075

[B52] Moskowitz M. A. Lo E. H. Iadecola C. (2010). The Science of Stroke: Mechanisms in Search of Treatments. Neuron 67, 181–198. 10.1016/j.neuron.2010.07.002 20670828 PMC2957363

[B53] Ngowi E. E. Wang Y.-Z. Qian L. Helmy Y. A. S. H. Anyomi B. Li T. (2021). The Application of Nanotechnology for the Diagnosis and Treatment of Brain Diseases and Disorders. Front. Bioeng. Biotechnol. 9, 629832. 10.3389/fbioe.2021.629832 33738278 PMC7960921

[B54] Obeso J. A. Stamelou M. Goetz C. G. Poewe W. Lang A. E. Weintraub D. (2017). Past, Present, and Future of Parkinson's Disease: A Special Essay on the 200th Anniversary of the Shaking Palsy. Mov. Disord. 32, 1264–1310. 10.1002/mds.27115 28887905 PMC5685546

[B55] OHSU (2022). MR, Histologic and EM Imaging of Intravenous Ferumoxytol in Central Nervous System (CNS) Inflammation.

[B56] Orendorff R. Peck A. J. Zheng B. Shirazi S. N. Matthew Ferguson R. Khandhar A. P. (2017). Firstin Vivotraumatic Brain Injury Imaging via Magnetic Particle Imaging. Phys. Med. Biol. 62, 3501–3509. 10.1088/1361-6560/aa52ad 28378708 PMC5736300

[B57] Paefgen V. Doleschel D. Kiessling F. (2015). Evolution of Contrast Agents for Ultrasound Imaging and Ultrasound-Mediated Drug Delivery. Front. Pharmacol. 6, 197. 10.3389/fphar.2015.00197 26441654 PMC4584939

[B58] Patriarchi T. Cho J. R. Merten K. Howe M. W. Marley A. Xiong W.-H. (2018). Ultrafast Neuronal Imaging of Dopamine Dynamics with Designed Genetically Encoded Sensors. Science 360. 10.1126/science.aat4422 PMC6287765 29853555

[B59] Pongrac I. M. Ahmed L. B. Mlinarić H. Jurašin D. D. Pavičić I. Marjanović Čermak A. M. (2018). Surface Coating Affects Uptake of Silver Nanoparticles in Neural Stem Cells. J. Trace Elem. Med. Biol. 50, 684–692. 10.1016/j.jtemb.2017.12.003 29273317

[B60] Rivet C. J. Yuan Y. Borca-Tasciuc D.-A. Gilbert R. J. (2012). Altering Iron Oxide Nanoparticle Surface Properties Induce Cortical Neuron Cytotoxicity. Chem. Res. Toxicol. 25, 153–161. 10.1021/tx200369s 22111864 PMC4183191

[B61] Sarin H. Kanevsky A. S. Wu H. Sousa A. A. Wilson C. M. Aronova M. A. (2009). Physiologic Upper Limit of Pore Size in the Blood-Tumor Barrier of Malignant Solid Tumors. J. Transl. Med. 7, 51. 10.1186/1479-5876-7-51 19549317 PMC2706803

[B62] Sharma A. K. Schultz J. W. Prior J. T. Rath N. P. Mirica L. M. (2017). Coordination Chemistry of Bifunctional Chemical Agents Designed for Applications in 64Cu PET Imaging for Alzheimer's Disease. Inorg. Chem. 56, 13801–13814. 10.1021/acs.inorgchem.7b01883 29112419 PMC5698879

[B63] Sillerud L. O. Solberg N. O. Chamberlain R. Orlando R. A. Heidrich J. E. Brown D. C. (2013). SPION-enhanced Magnetic Resonance Imaging of Alzheimer's Disease Plaques in AβPP/PS-1 Transgenic Mouse Brain. Jad 34, 349–365. 10.3233/JAD-121171 23229079 PMC4801216

[B64] Sim S. Wong N. (2021). Nanotechnology and its Use in Imaging and Drug Delivery (Review). Biomed. Rep. 14, 1–9. 10.3892/br.2021.1418 33728048 PMC7953199

[B65] Sirsi S. R. Borden M. A. (2009). Microbubble Compositions, Properties and Biomedical Applications. Bubble Sci. Eng. Technol. 1, 3–17. 10.1179/175889709X446507 20574549 PMC2889676

[B66] Smits L. P. Coolen B. F. Panno M. D. Runge J. H. Nijhof W. H. Verheij J. (2016). Noninvasive Differentiation between Hepatic Steatosis and Steatohepatitis with MR Imaging Enhanced with USPIOs in Patients with Nonalcoholic Fatty Liver Disease: A Proof-Of-Concept Study. Radiology 278, 782–791. 10.1148/radiol.2015150952 26348104

[B67] Stark D. D. Weissleder R. Elizondo G. Hahn P. F. Saini S. Todd L. E. (1988). Superparamagnetic Iron Oxide: Clinical Application as a Contrast Agent for MR Imaging of the Liver. Radiology 168, 297–301. 10.1148/radiology.168.2.3393649 3393649

[B68] Surmeier D. J. Obeso J. A. Halliday G. M. (2017). Selective Neuronal Vulnerability in Parkinson Disease. Nat. Rev. Neurosci. 18, 101–113. 10.1038/nrn.2016.178 28104909 PMC5564322

[B69] Triantafyllou M. Studer U. E. Birkhäuser F. D. Fleischmann A. Bains L. J. Petralia G. (2013). Ultrasmall Superparamagnetic Particles of Iron Oxide Allow for the Detection of Metastases in Normal Sized Pelvic Lymph Nodes of Patients with Bladder And/or Prostate Cancer. Eur. J. Cancer 49, 616–624. 10.1016/j.ejca.2012.09.034 23084842

[B70] Vallabani N. V. S. Singh S. Karakoti A. S. (2019). Magnetic Nanoparticles: Current Trends and Future Aspects in Diagnostics and Nanomedicine. Cdm 20, 457–472. 10.2174/1389200220666181122124458 30465498

[B71] Varallyay C. G. Nesbit E. Fu R. Gahramanov S. Moloney B. Earl E. (2013). High-resolution Steady-State Cerebral Blood Volume Maps in Patients with Central Nervous System Neoplasms Using Ferumoxytol, a Superparamagnetic Iron Oxide Nanoparticle. J. Cereb. Blood Flow. Metab. 33, 780–786. 10.1038/jcbfm.2013.36 23486297 PMC3653563

[B72] Viola K. L. Sbarboro J. Sureka R. De M. Bicca M. A. Wang J. (2015). Towards Non-invasive Diagnostic Imaging of Early-Stage Alzheimer's Disease. Nat. Nanotech 10, 91–98. 10.1038/nnano.2014.254 PMC4300856 25531084

[B73] Wang J. Ni D. Bu W. Zhou Q. Fan W. Wu Y. (2015). BaHoF 5 Nanoprobes as High-Performance Contrast Agents for Multi-Modal CT Imaging of Ischemic Stroke. Biomaterials 71, 110–118. 10.1016/j.biomaterials.2015.08.038 26321059

[B74] Wang T. Hou Y. Bu B. Wang W. Ma T. Liu C. (2018). Timely Visualization of the Collaterals Formed during Acute Ischemic Stroke with Fe3 O4 Nanoparticle-Based MR Imaging Probe. Small 14, 1800573. 10.1002/smll.201800573 29665290

[B75] Wilhelm S. Anthony J. T. Qin D. Seiichi O. Julie A. Harold F. D. (2016). Analysis of Nanoparticle Delivery to Tumours. Nat. Rev. Mat. 1, 1–12. 10.1038/natrevmats.2016.14

[B76] Xu F. Piett C. Farkas S. Qazzaz M. Syed N. I. (2013). Silver Nanoparticles (AgNPs) Cause Degeneration of Cytoskeleton and Disrupt Synaptic Machinery of Cultured Cortical Neurons. Mol. Brain 6, 29. 10.1186/1756-6606-6-29 23782671 PMC3695839

[B77] Zhang L. Gu F. Chan J. Wang A. Langer R. Farokhzad O. (2008). Nanoparticles in Medicine: Therapeutic Applications and Developments. Clin. Pharmacol. Ther. 83, 761–769. 10.1038/sj.clpt.6100400 17957183

[B78] Zhou Z. Bai R. Wang Z. Bryant H. Lang L. Merkle H. (2019). An Albumin-Binding T1-T2 Dual-Modal MRI Contrast Agents for Improved Sensitivity and Accuracy in Tumor Imaging. Bioconjugate Chem. 30, 1821–1829. 10.1021/acs.bioconjchem.9b00349 31117347

